# Does Cataract Surgery Alleviate Poverty? Evidence from a Multi-Centre Intervention Study Conducted in Kenya, the Philippines and Bangladesh

**DOI:** 10.1371/journal.pone.0015431

**Published:** 2010-11-09

**Authors:** Hannah Kuper, Sarah Polack, Wanjiku Mathenge, Cristina Eusebio, Zakia Wadud, Mamunur Rashid, Allen Foster

**Affiliations:** 1 Department of Epidemiology and Population Health, London School of Hygiene and Tropical Medicine, London, United Kingdom; 2 Department of Infectious and Tropical Diseases, London School of Hygiene & Tropical Medicine, London, United Kingdom; 3 Cataract Foundation of the Philippines, Bacolod, Negros Occidental, Philippines; 4 Child Sight Foundation, Dhaka, Bangladesh; 5 CSS Rawm Hospital, Khulna, Bangladesh; University of KwaZulu Natal, South Africa

## Abstract

**Background:**

Poverty and blindness are believed to be intimately linked, but empirical data supporting this purported relationship are sparse. The objective of this study is to assess whether there is a reduction in poverty after cataract surgery among visually impaired cases.

**Methodology/Principal Findings:**

A multi-centre intervention study was conducted in three countries (Kenya, Philippines, Bangladesh). Poverty data (household per capita expenditure – PCE, asset ownership and self-rated wealth) were collected from cases aged ≥50 years who were visually impaired due to cataract (visual acuity<6/24 in the better eye) and age-sex matched controls with normal vision. Cases were offered free/subsidised cataract surgery. Approximately one year later participants were re-interviewed about poverty. 466 cases and 436 controls were examined at both baseline and follow-up (Follow up rate: 78% for cases, 81% for controls), of which 263 cases had undergone cataract surgery (“operated cases”). At baseline, operated cases were poorer compared to controls in terms of PCE (Kenya: $22 versus £35 p = 0.02, Bangladesh: $16 vs $24 p = 0.004, Philippines: $24 vs 32 p = 0.0007), assets and self-rated wealth. By follow-up PCE had increased significantly among operated cases in each of the three settings to the level of controls (Kenya: $30 versus £36 p = 0.49, Bangladesh: $23 vs $23 p = 0.20, Philippines: $45 vs $36 p = 0.68). There were smaller increases in self-rated wealth and no changes in assets. Changes in PCE were apparent in different socio-demographic and ocular groups. The largest PCE increases were apparent among the cases that were poorest at baseline.

**Conclusions/Significance:**

This study showed that cataract surgery can contribute to poverty alleviation, particularly among the most vulnerable members of society. This study highlights the need for increased provision of cataract surgery to poor people and shows that a focus on blindness may help to alleviate poverty and achieve the Millennium Development Goals.

## Introduction

Poverty and blindness are believed to be intimately linked, with poverty predisposing to blindness, and blindness exacerbating poverty by limiting employment opportunities, or by incurring treatment cost. However, empirical data supporting these claims are sparse, particularly for low and middle income countries (LMICs) [1]–[3], and few data are available showing the impact of measures to alleviate blindness on poverty reduction [4]. This information is needed urgently as improvements in health and reductions in poverty are central to the achievement of the Millennium Development Goals (MDG).

Cataract is the leading cause of blindness globally, affecting almost 18 million people [5]. Cataract surgery is a relatively cheap and highly cost-effective intervention, even in LMICs [6]. However, competing financial demands limits the allocation of funds to cataract surgery both at the national and individual level, so that poor people may be less likely to have their cataract treated. Blindness may also exacerbate poverty by reducing opportunities of the individual or their families to engage in income generating activities [4]. Consequently, cataract blindness is markedly more prevalent in LMICs [5], and may be more common among poor people within countries [1]–[3], although data are sparse. This relationship with poverty is also evident for disability in general [7], although few studies have attempted to unravel the nature of the relationship between poverty and cataract or other types of disability through empirical data.

The Cataract Impact Study was undertaken to assess the impact of cataract visual impairment and cataract surgery on poverty, activities and quality of life in three low income countries [8]–[11]. Our baseline analyses showed that cases visually impaired from cataract were significantly poorer, less likely to be involved in productive activities and had worse quality of life than controls with normal vision in Kenya, the Philippines and Bangladesh [8]–[11]. At follow-up the cases who had undergone cataract surgery had significantly improved quality of life [12], were significantly more likely to participate in productive activities and received less assistance from caregivers [13]. We hypothesise that these changes would have resulted in reduced poverty among the operated cases at follow-up. The aim of the current analysis is to assess the impact of cataract surgery on poverty among cases with cataract in these three settings.

## Methods

### Ethics statement

Informed signed or thumb-printed consent was obtained from all cases and controls. All cases with operable cataract were referred for surgery. Ethical approval was obtained from the ethics committees of the London School of Hygiene & Tropical Medicine, the Kenya Medical Research Institute, the Bangladesh Medical Research Council and the University of St. La Salle, Bacolod, Philippines.

### Study overview

The ‘Cataract Impact Study’ was a longitudinal intervention study conducted in Kenya (Nakuru district), Bangladesh (Satkhira district) and the Philippines (Negros Island and Antique district) [8]–[13]. At baseline cases with visual impairment from cataract and controls without visual impairment were identified and interviewed about time-use, health related quality of life and poverty. All cases were offered free or subsidized surgery. Approximately one year later cases and controls were re-traced, re-examined and re-interviewed. This paper presents the findings from the poverty data.

### Participants

Sample size calculations were powered to detect a 30% improvement in per capita expenditure one year after cataract surgery, with an alpha of 0.05 and 80% power and necessitated a sample of 100 cases examined at baseline and follow-up in each country. A total of 180 cases were required at baseline in each country, assuming that 75% of cases underwent surgery and 75% were followed at one year.

Cases and controls were identified primarily through population-based blindness surveys undertaken at baseline [14]–[16]. Clusters of 50 people aged ≥50 years were selected through probability-proportionate to size sampling, using either the census (Philippines and Bangladesh) or electoral role (Kenya) as the sampling frame. Households within clusters were selected through a modification of compact segment sampling, whereby a map was drawn of the enumeration area which was divided into segments each including approximately 50 people aged ≥50 years and one segment was chosen at random [17]. Households in the segment were included sequentially until 50 people aged ≥50 years were identified. The surveys included 3503 (93% response rate) people aged ≥50 years in Kenya, 4868 (92%) in Bangladesh, 2774 (76%) in Negros and 3177 (83%) in Antique.

All people aged ≥50 years underwent visual acuity (VA) testing and ophthalmic examination. VA was measured in full daylight with available spectacle correction with a Snellen tumbling “E” chart using optotype size 6/18 (20/60) on one side and size 6/60 (20/200) on the other side at 6 or 3 metre distance. If the VA was <6/18 in either eye then pinhole vision was also measured. Participants with pinhole vision <6/18 but >6/60 in the better eye due to age-related cataract were given a second VA test using an ‘E’ of size 6/24. The ophthalmologist examined all eyes with a presenting VA<6/18 with a torch, direct ophthalmoscope and/or portable slit lamp.

Cases were invited for participation if they had pinhole VA <6/24 in the better eye due to cataract, as diagnosed by an ophthalmologist. One (or up to two in Bangladesh) age- sex- cluster-matched controls without visual impairment (presenting VA>6/18 in better eye) were selected per case from the eligible adults examined in the same cluster. During the survey the eligible controls in each cluster were listed by gender and age group (50–54, 55–59, 65–69 and ≥70 years). When a case was identified, one control (or up to two in Bangladesh) of the same gender and age group was selected at random for inclusion by drawing lots. If there were no matching control at that time then the next eligible control identified in the cluster was recruited. Cases and controls who were significantly communication impaired (e.g. deaf) were excluded (<5 per country).

Due to logistical and time constraints, additional cases were identified in each setting through community-based case detection. In Kenya and Negros (Philippines) additional clusters were selected using probability proportionate to size sampling after completion of the population based-survey. These clusters were visited in advance and asked that all people aged ≥50 years with eyesight problems come to a central point on a specified day and that people unable to attend (e.g. due to blindness or physical disability) be noted. All people attending the central point and those unable to leave their households underwent an eye examination using the procedures described above. People who met the case definition were invited to participate in the study and were interviewed in their homes. In Bangladesh and Antique (Philippines), case finding was conducted simultaneously with the survey so that age- gender matched controls were also included for these cases. In each cluster the teams asked to be taken to a community member with eye problems living within the boundaries of the cluster but outside the selected segment. The ophthalmologist conducted the ophthalmic examination at the household to identify eligible cases.

### Intervention

In Kenya and Bangladesh all cases were offered free cataract surgery at the local hospital, with free transport. In the Philippines, patients were referred for surgery which was subsidised for patients who could not afford the fee. “Operated cases” were those who accepted the surgery while “un-operated cases” did not.

### Data collection

Baseline surveys were conducted between January 2005 and May 2006. Follow up surveys were undertaken approximately one year later, during the same climatic season as the baseline. Interviews were conducted in respondents' own homes by trained interviewers who were regularly observed by supervisors.

### Measures of poverty

The person primarily responsible for household finances was interviewed to assess poverty as measured through (a) household PCE to indicate consumption, (b) asset ownership and (c) self rated wealth:

- PCE: the household informant was asked to recall the monetary value of food that was purchased, consumed from home production, received as payment in kind or as gifts over the last month by all household members (not including those away) [18]. They were also questioned about expenditure on education, health, household and personal items and rent paid (or rental equivalent for home owners). Consumption was assessed over the previous one week period for frequently consumed items, and this was scaled up to estimate monthly consumption. The amount consumed over the previous month was assessed for items that were consumed more rarely. In total, 85 items were included in the questionnaire in Kenya, 90 in the Philippines and 79 in Bangladesh. The consumption on all items was summed to calculate total monthly household consumption, and this was converted to US dollars at the average exchange rate between baseline and follow-up ($1 = 74 shillings, 67 taka, 51 peso). PCE was calculated by dividing total monthly household consumption by the number of household members.
- Asset ownership: The informant was also asked about the number and type of context-specific assets owned by the household, including furniture items, electrical equipment, cattle and vehicles. Information was collected on household characteristics (e.g. building material of the floor, roof and walls, type of toilet and the number of rooms). This information was used to derive a relative index of household assets using Principle Components Analysis (PCA) [19].
- Self-rated wealth: participants were asked *“On a scale of 1 to 10, how well-off do you think your household is in relation to the other households in the village?”*.

### Covariates

Cases and controls were interviewed about standard socio-demographic indicators, including household composition, education, and employment. They were also asked about, self-rated health (ranking their “health state today” on a scale of 1–100), and time-use as described in detail elsewhere [10], [13], [20].

### Questionnaire development and training

The questionnaires were translated into the local languages (3 in Kenya, 3 in the Philippines and 1 in Bangladesh) and back-translated by independent translators who also commented on appropriateness of language used. The questionnaire was reviewed and pilot tested in each setting and small modifications were made, where appropriate, to ensure local understanding. Interviewers were trained for one week at baseline and at follow-up.

### Statistical analysis

Data on household expenditure were cleaned, excluding gross outliers and imputing rental equivalents based on household characteristics and non-rent expenditure where these estimates were missing or unreasonably low (<$1 per month – 34 in total). PCE and asset scores were divided into quartiles for each country, based on the distribution of the data for the cases and controls combined. All data analyses were restricted to participants with both baseline and follow-up data and were conducted for each country separately.

The following analyses were undertaken:

- Comparison of socio-demographic characteristics between cases and controls: The baseline socio-demographic characteristics of operated cases and controls were compared calculating p-values through t-test for continuous variables and chi-square for discrete variables (or exact test if cell count<5). We also compared characteristics of un-operated cases to operated cases.
- Assessment of differences in poverty between cases and controls: We compared both baseline and follow up scores for PCE, assets and self-rated wealth between operated cases and controls using the t-test for log-transformed PCE scores and assets, and the Mann-Witney test for household rank. We also compared scores of un-operated cases to operated cases.
- Assessment of change in poverty over time: We compared baseline and follow-up scores for the three poverty variables separately for operated cases, un-operated cases and operated controls, calculating paired t-test derived p-values for PCE (all countries) and assets (Bangladesh and Philippines), and Wilcoxon signed rank test for household rank and assets scores in Kenya (these data were skewed and could not be transformed).
- Multivariate analyses comparing poverty in operated cases and controls: Logistic regression analyses were undertaken, separately for each country, comparing operated cases and controls for the three poverty variables divided into quartiles at both baseline and at follow-up, adjusting analyses for the matching variables (age, sex and location, and study site in the Philippines). Analyses were additionally adjusted for social support indicators (marital status and household size), self-rated health and markers of early life poverty (school attendance and literacy), as potential confounders.
- Identification of predictors of change in PCE among operated cases: We compared the mean change in PCE between baseline and follow-up among operated cases stratified by socio-demographic and ocular groups, calculating t-test derived p-values for comparing log PCE values for baseline and follow-up and comparing change between the groups. We also compared the mean allocation to different expenditure categories (e.g. food, education health etc) at baseline and at follow-up among operated cases.

## Results

At baseline we included 142 cases and 75 controls in Kenya, 217 cases and 280 controls in Bangladesh and 240 cases and 182 controls in the Philippines (Table 1). Uptake of surgery among cases was consistently low (Kenya: 58%, Bangladesh: 54%, Philippines: 47%). Follow-up rates were high, particularly for the operated cases (>79%) and controls (>75%). Overall, 62% of loss to follow-up was due to drop-out, 34% due to death, and 4% due to refusal/inability to communicate. Operated cases, un-operated cases and controls lost to follow-up did not differ systematically from those included in terms of socio-demographic characteristics (data not shown).

**Table 1:** Follow-up by country for operated cases, controls and un-operated cases.

Country	Participant type	Total at Baseline (N)	Examined at follow-up (%)	Reasons for loss to follow-up (%)		
				Lost	Died	Refused/unable to communicate
Kenya	Operated cases	82	65 (79%)	9 (53%)	8 (47%)	0 (0%)
	Controls	75	56 (75%)	16 (84%)	3 (16%)	0 (0%)
	Un-operated cases	60	40 (67%)	12 (60%)	7 (35%)	1 (5%)
Bangladesh	Operated cases	117	99 (85%)	11 (61%)	6 (33%)	1 (6%)
	Controls	280	223 (80%)	46 (81%)	10 (18%)	1 (2%)
	Un-operated cases	100	70 (70%)	20 (67%)	7 (23%)	3 (10%)
Philippines	Operated cases	113	99 (88%)	7 (50%)	7 (50%)	0 (0%)
	Controls	182	157 (86%)	18 (72%)	6 (24%)	1 (4%)
	Un-operated cases	127	93 (73%)	12 (35%)	20 (59%)	2 (6%)

More of the cases observed at baseline and follow-up were identified from the population-based survey (Kenya = 60, Bangladesh = 125, Philippines = 113), than through population based case finding (Kenya = 44, Bangladesh = 41, Philippines = 78). In Kenya and the Philippines there was no difference between these two case types in socio-demographic characteristics (age, sex, marital status, literacy, job, VA). In Bangladesh RAAB cases were significantly older and had better VA. All controls were identified through the population-based survey.

### Comparison of baseline socio-demographic characteristics

Operated cases and controls were broadly similar in age, sex and marital status in the three countries, although operated cases were slightly older than controls in Bangladesh (Table 2). Controls were far more likely to have a job at baseline than cases and to be literate, except in the Philippines. Baseline self-rated health was consistently higher among controls compared to operated cases. Operated cases were also compared to un-operated cases. The un-operated cases were older. In Bangladesh the un-operated cases were more likely to be female and in Kenya and Bangladesh they were less likely to be married than operated cases, but they were similar in terms of job status and literacy. Baseline self-rated health was lower for un-operated cases compared to operated cases in Bangladesh and the Philippines, and in Kenya the operated cases had poorer baseline vision than un-operated cases.

**Table 2:** Baseline demographic characteristics of cases and controls examined at baseline and follow-up.

Variable	Kenya					Bangladesh					Philippines				
	Operated cases (n = 65)	Unoperated Cases (n = 39)	Controls (n = 55)	P-value operated vs un-operated cases	P-value operated cases vs controls	Operated cases (n = 99)	Unoperated Cases (n = 67)	Controls (n = 221)	P-value operated vs un-operated cases	P-value operated cases vs controls	Operated cases (n = 99)	Unoperated Cases (n = 92)	Controls (n = 152)	P-value operated vs un-operated cases	P-value operated cases vs controls
Mean age (95% CI)	78 (75–80)	82 (79–84)	75 (73–78)	0.03	0.15	71 (69–73)	75 (72–77)	69 (68–70)	0.04	0.03	72 (70–73)	77 (75–78)	71 (70–73)	0.0002	0.85
Female	49%	64%	51%	0.14	0.85	53%	72%	57%	0.01	0.46	62%	70%	56%	0.25	0.37
Married	56%	28%	61%	0.007	0.54	48%	32%	58%	0.03	0.12	45%	46%	56%	0.92	0.09
Literate	29%	31%	56%	0.87	0.003	8%	9%	28%	0.84	<0.0001	15%	22%	12%	0.24	0.44
Job	0%	0%	7%	NA	0.01*	6%	12%	18%	0.18	0.006	9%	3%	17%	0.14*	0.09
Mean self-rated health (95% CI)	50 (45–54)	52 (46–57)	58 (53–63)	0.62	0.04	50 (45–55)	42 (37–47)	59 (56–62)	0.04	0.0006	53 (51–56)	49 (47–52)	62 (59–65)	0.05	<0.0001
Pre op VA															
Normal	0	0	100%	0.008	NA	0	0	100%	0.69	NA	0	0	100%	0.18	NA
VI	40%	56%				29%	22%				26%	34%			
SVI	18%	33%				16%	21%				21%	14%			
Blind	18%	3%				12%	15%				24%	31%			
PL	23%	8%				42%	42%			29%	20%				

*Fisher's exact test.

### Assessment of differences in poverty between cases and controls and change over time

Both cases and controls were generally poor at baseline and remained so at follow-up, with daily PCE averaging $0.53–$1.50 per person. At baseline, operated cases had significantly lower PCE compared to controls in all three countries (Table 3). At follow up, average PCE had increased among operated cases by $8 in Kenya (36%, p = 0.07), $7 in Bangladesh (44%, p<0.0001) and $21 in the Philippines (88%, p<0.0001) and was no longer significantly lower than among controls.

**Table 3:** Comparison of PCE, assets and household rank at baseline and follow up for operated cases and controls.

Poverty measures	Mean scores (95% CI)	Kenya			Bangladesh			Philippines		
		Operated cases (n = 65)	Controls (n = 56)	p-value operated cases versus controls	Operated cases (n = 99)	Controls (n = 222)	p-value operated cases versus controls	Operated cases (n = 99)	Controls (n = 152)	p-value operated cases versus controls
**PCE ($)**	Baseline	22 (18–26)	35 (24–46)	0.02	16 (12–21)	24 (16–33)	0.004	24 (19–28)	32 (27–38)	0.0007
	Follow-up	30 (22–37)	36 (22–51)	0.49	23 (18–28)	23 (21–24)	0.20	45 (28–62)	36 (30–42)	0.68
	Change (95% CI)	8 (0–15)	1 (−6–9)		7 (1–12)	−2 (−10–7)		21 (4–38)	3 (−4–10)	
	p-value for change	0.07	0.71		<0.0001	<0.0001		<0.0001	0.22	
**Assets**	Baseline	−0.8 (−1.2–−0.3)	0.5 (−0.3–1.3)	0.004	−0.7 (−1.1–−0.2)	0.2 (−0.1–0.6)	0.003	−0.2 (−0.7–0.3)	0.3 (−0.2–0.7)	0.14
	Follow-up	−0.8 (−1.2–−0.4)	0.2 (−0.5–1.0)	0.02	−0.7 (−1.1–−0.2)	0.2 (−0.1–0.5)	0.002	−0.1 (−0.6–0.4)	0.4 (0–0.8)	0.16
	Change (95% CI)	0 (−0.2–0.3)	−0.3 (−0.5–0)		0.03 (−0.1–0.2)	0 (−0.1–0.1)		0.1 (−0.2–0.5)	0.1 (−0.1–0.3)	
	p-value for change	0.36	0.05		0.81	0.96		0.47	0.35	
**Household rank**	Baseline	3.4 (3.1–3.8)	4.9 (4.4–5.4)	<0.0001	3.8 (3.4–4.2)	4.5 (4.2–4.8)	0.003	4.1 (3.8–4.4)	4.3 (4.0–4.6)	0.31
	Follow-up	4.1 (3.7–4.5)	4.8 (4.3–5.3)	0.05	3.9 (3.5–4.3)	4.4 (4.1–4.6)	0.02	4.6 (4.3–4.9)	4.6 (4.3–4.9)	0.83
	Change (95% CI)	0.7 (0.2–1.1)	−0.1 (−0.7–0.5)		0.1 (−0.2–0.4)	−0.1 (−0.4–0.1)		0.5 (0.1–0.9)	0.3 (−0.3–0.5)	
	p-value for change	0.005	0.80		0.64	0.61		0.007	0.08	

Baseline mean asset scores were also poorer among operated cases than controls in each country (Kenya: −0.8 vs 0.5; Bangladesh; −0.7 vs 0.2), although this was non-significant in the Philippines (−0.2 vs 0.3). There was virtually no change in asset scores among either operated cases or controls between baseline and follow-up, so that controls retained higher asset scores in Kenya and Bangladesh.

Household rank was significantly lower among operated cases than controls at baseline in Kenya (3.4 vs 4.9) and Bangladesh (3.8 vs 4.5), but not in the Philippines (4.1 vs 4.3). At follow up, household rank had increased significantly among operated cases in Kenya and the Philippines compared to baseline, but remained significantly lower than controls in both Kenya and Bangladesh and no different in the Philippines.

PCE, assets and household rank remained broadly similar between baseline and follow-up among controls.

Un-operated cases were similar to operated cases at baseline in terms of PCE (Kenya $20.2, p = 0.59; Bangladesh $19.5, p = 0.35, Philippines $27.3, p = 0.25) assets (−1.0, p = 0.58; −0.21, p = 0.29; −0.3, p = 0.76) and household rank (3.2, p = 0.46; 4.1, p = 0.38; 4.0, p = 0.68). There were significant, though generally smaller, increases in PCE among un-operated cases between baseline and follow up in Kenya ($8 increase, 95% CI = $0–17 p = 0.05), Bangladesh ($3, −$2–$8 p = 0.02) and the Philippines ($7, $0–14 p = 0.01). There was generally no change at follow-up in assets (Kenya: −0.8, Philippines −0.6) or self-rated wealth (Kenya: 3.7; Bangladesh: 4.0, Philippines: 4.2), and only the increase for assets in Bangladesh reached statistical significance (0.3 points, 0.1–0.6 p = 0.01).

### Multivariate analyses of the association between poverty and case/control status at baseline and follow-up

We divided poverty indicator scores into quartiles to allow a comparison of operated cases and controls with adjustment for potential confounders. At baseline, operated cases were significantly more likely than controls to be in the poorest quartile of PCE in Kenya (OR = 3.3, 95% CI = 1.0–10.8), Bangladesh (3.2, 1.5–6.6) and the Philippines (4.4, 1.9–10.0), and there was a significant trend of association between falling PCE and case status (Table 4). At follow up, these differences were no longer statistically significant in Kenya (1.1, 0.3–3.2) and the Philippines (1.1, 0.5–2.4) and were weakened in Bangladesh (2.2, 1.1–4.5). Similarly, at baseline operated cases were much more likely than controls to be in the poorest quartile for asset ownership and household rank. The associations between case status and asset ownership or household rank were weaker at follow-up, particularly in Kenya and the Philippines. For all measures of poverty, the trends of level of poverty in relation to case status remained statistically significant in Bangladesh at follow-up but not in Kenya or the Philippines. Additional adjustment for marital status, household size, baseline self rated health, school attendance and literacy weakened the associations at baseline, though they generally remained strong and statistically significant, and did not change the follow-up associations (data not shown).

**Table 4:** Multivariable analyses for the comparison of poverty variables at baseline and follow-up among operated cases and controls.

Poverty measures	Quartiles	Kenya		Bangladesh		Philippines	
		Baseline	Follow-up	Baseline	Follow-up	Baseline	Follow-up
		OR (95% CI) adjusted for age, sex and location	OR (95% CI) adjusted for age, sex and location	OR (95% CI) adjusted for age, sex and location	OR (95% CI) adjusted for age, sex and location	OR (95% CI) adjusted for age, sex, location and province	OR (95% CI) adjusted for age, sex, location and province
Per capita expenditure	1 (lowest)	3.3 (1.0–10.8)	1.1 (0.3–3.2)	3.2 (1.5–6.6)	2.2 (1.1–4.5)	4.4 (1.9–10.0)	1.1 (0.5–2.4)
	2	3.5 (1.1–11.5)	0.6 (0.2–1.9)	1.7 (0.8–3.5)	1.1 (0.5–2.1)	4.4 (1.9–10.2)	0.8 (0.4–1.7)
	3	1.1 (0.4–3.4)	1.5 (0.5–4.4)	1.7 (0.8–3.7)	1.1 (0.5–2.2)	1.7 (0.7–3.8)	0.6 (0.3–1.3)
	4 (highest)	Baseline	Baseline	Baseline	Baseline	Baseline	Baseline
	P for trend	0.01	0.69	0.003	0.03	<0.0001	0.66
Assets	1 (lowest)	4.7 (1.4–16.6)	2.6 (0.8–8.6)	3.0 (1.4–6.2)	2.2 (1.1–4.5)	1.7 (0.7–3.8)	1.7 (0.8–3.9)
	2	4.3 (1.3–14.4)	3.8 (1.1–13.5)	2.1 (1.0–4.4)	1.6 (0.8–3.3)	2.2 (1.0–5.0)	1.6 (0.7–3.5)
	3	3.2 (1.0–10.6)	2.2 (0.7–7.1)	1.7 (0.8–3.5)	1.0 (0.5–2.1)	2.2 (1.0–4.8)	1.6 (0.7–3.4)
	4 (highest)	Baseline	Baseline	Baseline	Baseline	Baseline	Baseline
	P for trend	0.01	0.11	0.003	0.01	0.23	0.20
Household rank	1 (lowest)	11.2 (2.6–48.3)	2.2 (0.6–8.3)	3.0 (1.4–6.4)	2.4 (1.1–5.2)	2.1 (0.8–5.9)	0.9 (0.4–2.1)
	2	8.4 (2.2–32.1)	4.4 (1.4–13.9)	2.1 (1.0–4.3)	1.5 (0.8–3.1)	2.7 (0.9–7.8)	1.5 (0.7–3.4)
	3	2.8 (0.7–10.8)	3.6 (1.2–11.1)	1.6 (0.7–3.4)	1.5 (0.7–3.3)	1.2 (0.5–3.2)	0.8 (0.3–1.6)
	4 (highest)	Baseline	Baseline	Baseline	Baseline	Baseline	Baseline
	P for trend	0.0002	0.13	0.003	0.04	0.05	0.73

Operated cases were also compared to un-operated cases for the three poverty indicators at baseline and follow-up (Table 5). In Kenya, there was no difference between operated and un-operated cases in PCE or assets at either baseline or follow-up. At baseline, there was no difference in household rank, but at follow-up the operated cases were less likely to be in the poorest quartiles of household rank compared to the un-operated cases. In Bangladesh, the operated cases were somewhat poorer than the un-operated cases at baseline in terms of PCE, but this association disappeared at follow-up. Assets and household rank did not differ between operated and un-operated cases, either at baseline or follow-up. In the Philippines the operated and un-operated cases were similar at baseline for all three poverty measures. At follow-up, the operated cases were less likely than the un-operated cases to be in the poorest quartiles for each of the three measures.

**Table 5:** Multivariable analyses for the comparison of poverty variables at baseline and follow-up among operated cases and unoperated cases.

Poverty measures	Quartiles	Kenya		Bangladesh		Philippines	
		Baseline	Follow-up	Baseline	Follow-up	Baseline	Follow-up
		OR (95% CI) adjusted for age, sex and location	OR (95% CI) adjusted for age, sex and location	OR (95% CI) adjusted for age, sex and location	OR (95% CI) adjusted for age, sex and location	OR (95% CI) adjusted for age, sex, location and province	OR (95% CI) adjusted for age, sex, location and province
Per capita expenditure	1 (lowest)	0.8 (0.3–2.6)	0.8 (0.3–2.2)	2.5 (1.0–6.3)	0.8 (0.3–2.0)	1.3 (0.5–3.0)	0.5 (0.2–1.2)
	2	0.6 (0.2–1.8)	0.7 (0.2–2.0)	1.9 (0.7–5.0)	0.6 (0.2–1.7)	1.8 (0.7–4.3)	0.5 (0.2–1.3)
	3	1.4 (0.4–4.7)	1.8 (0.5–5.9)	1.8 (0.7–4.8)	0.5 (0.2–1.3)	2.3 (0.8–6.2)	0.4 (0.2–0.9)
	4 (highest)	Baseline	Baseline	Baseline	Baseline	Baseline	Baseline
	P for trend	0.48	0.35	0.08	0.95	0.87	0.24
Assets	1 (lowest)	0.8 (0.3–2.6)	0.9 (0.3–2.9)	1.1 (0.4–2.8)	1.0 (0.4–2.5)	0.6 (0.2–1.6)	0.4 (0.2–1.1)
	2	1.6 (0.4–6.1)	0.9 (0.3–2.9)	0.9 (0.3–2.3)	1.0 (0.4–2.6)	0.8 (0.3–2.0)	0.6 (0.2–1.4)
	3	2.1 (0.6–7.6)	1.9 (0.5–6.5)	2.0 (0.7–5.8)	1.5 (0.5–4.3)	1.2 (0.5–2.9)	0.6 (0.2–1.5)
	4 (highest)	Baseline	Baseline	Baseline	Baseline	Baseline	Baseline
	P for trend	0.37	0.52	0.62	0.78	0.22	0.09
Household rank	1 (lowest)	0.7 (0.1–4.2)	0.4 (0.1–1.8)	1.4 (0.5–3.5)	0.7 (0.3–2.0)	0.8 (0.3–2.5)	0.4 (0.2–1.1)
	2	1.2 (0.2–7.1)	0.5 (0.2–1.8)	2.1 (0.7–5.8)	1.8 (0.7–5.0)	0.9 (0.3–3.0)	1.0 (0.4–2.6)
	3	1.8 (0.3–12.9)	1.7 (0.4–6.4)	1.1 (0.4–3.0)	1.4 (0.4–4.2)	1.2 (0.4–3.5)	1.1 (0.4–2.6)
	4 (highest)	Baseline	Baseline	Baseline	Baseline	Baseline	Baseline
	P for trend	0.22	0.05	0.37	0.58	0.40	0.05

### Predictors of change among operated cases

**Figure 1:**
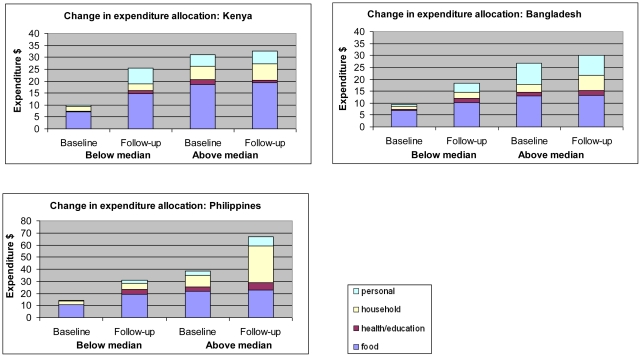
Change in allocation of expenses between baseline and follow-up among operated cases.

Predictors of change in PCE from baseline to follow-up were assessed among the operated cases (Table 6). There was no evidence for significant difference in change in PCE by any of the variables assessed, but some consistent trends were apparent. Improvements in PCE were larger among those <75 years compared to older participants in Bangladesh and the Philippines, and increases were larger among women than men in Kenya and the Philippines. Increases in PCE were consistently larger among unmarried than married people in all three countries. In the Philippines the increase in PCE was most apparent among people with high self-rated health or better baseline VA at baseline, while this was not apparent in Kenya and Bangladesh. No consistent trends were apparent for the change in PCE by outcome VA or number of eyes operated. In each setting the largest proportional increase in PCE was apparent among those in the poorer half of PCE at baseline compared to those above the median for PCE (Kenya: 158% increase in PCE versus 4% increase, Bangladesh: 94% versus 12%, Philippines: 116% versus 73%) (Figure 1).

**Table 6:** Predictors of change in PCE between baseline and follow-up among operated cases.

		Kenya				Bangladesh				Philippines			
		N	Baseline mean PCE (95% CI)	Follow up mean PCE (95% CI)	Mean change (95% CI)	N	Baseline mean PCE (95% CI)	Follow up mean PCE (95% CI)	Mean change (95% CI)	N	Baseline mean PCE (95% CI)	Follow up mean PCE (95% CI)	Mean change (95% CI)
Age	≤75	27	24 (17–32)	32 (22–43)	8 (−3–20)	64	15 (13–17)	23 (17–29)	8 (2–14)	56	20 (17–22)	43 (15–72)	24 (−5–52)
	>75	38	20 (15–26)	27 (17–37)	7 (−3–17)	35	19 (7–31)	23 (14–31)	4 (−8–16)	43	29 (19–38)	47 (33–60)	18 (3–33)
	p-value		0.34	0.30	0.88		0.92	0.80	0.51		0.11	0.03	0.72
Sex	Male	33	20 (15–24)	22 (17–27)	2 (−4–8)	47	18 (9–27)	25 (18–31)	7 (−3–16)	38	21 (16–25)	34 (22–47)	14 (3–25)
	Female	32	24 (16–32)	38 (24–51)	13 (0–27)	52	15 (12–18)	22 (15–28)	7 (0–13)	61	26 (19–32)	51 (25–78)	25 (−2–53)
	p-value		0.48	0.13	0.12		0.83	0.30	0.99		0.28	0.27	0.41
Married	Yes	35	20 (14–26)	24 (15–34)	4 (−8–16)	48	20 (11–28)	23 (18–27)	4 (−5–11)	43	22 (19–26)	35 (24–46)	13 (3–23)
	No	28	23 (16–30)	35 (24–46)	12 (3–21)	51	13 (11–15)	23 (15–31)	10 (2–18)	53	25 (17–33)	53 (23–84)	28 (−3–60)
	p-value		0.30	0.10	0.26		0.09	0.29	0.24		0.53	0.50	0.35
Literate	Yes	19	25 (18–32)	28 (19–36)	3 (−7–12)	8	17 (13–21)	25 (19–32)	8 (0–16)	84	24 (19–30)	49 (29–68)	24 (4–44)
	No	46	21 (15–26)	30 (21–40)	10 (0–19)	91	16 (12–21)	23 (18–28)	6 (0–13)	15	19 (15–24)	23 (13–33)	3 (−8–15)
	p-value		0.15	0.58	0.30		0.24	0.01	0.68		0.56	0.04	0.07
Baseline self-rated health	<median	19	17 (13–20)	24 (15–33)	8 (1–14)	47	15 (12–19)	23 (16–29)	8 (1–14)	59	25 (18–32)	33 (26–41)	8 (−1–18)
	≥median	46	24 (18–30)	32 (22–41)	7 (−2–17)	52	18 (10–26)	23 (16–30)	5 (−4–15)	40	22 (18–26)	62 (21–103)	40 (0–80)
	p-value		0.23	0.45	0.98		0.69	0.71	0.66		0.72	0.18	0.13
Baseline PCE	<median	28	10 (9–11)	26 (12–39)	16 (3–29)	59	9 (9–10)	18 (13–23)	9 (4–14)	61	14 (13–16)	31 (24–38)	17 (10–24)
	≥median	37	31 (25–37)	33 (24–41)	1 (−7–9)	40	27 (17–37)	30 (21–39)	3 (−9–16)	38	39 (29–49)	67 (24–110)	28 (−16–73)
	p-value		<0.0001	0.03	0.05		<0.001	0.0003	0.40		<0.0001	0.002	0.60
Baseline VA	MVI/SVI	38	20 (15–27)	28 (21–36)	8 (1–15)	45	15 (12–18)	20 (17–23)	5 (2–9)	47	24 (15–33)	53 (19–87)	29 (−6–64)
	Blind	27	24 (17–32)	31 (17–45)	7 (−8–22)	54	18 (10–25)	25 (17–34)	8 (−2–18)	52	23 (19–27)	37 (26–48)	14 (4–24)
	p-value		0.22	0.96	0.90		0.77	0.89	0.66		0.45	0.59	0.41
Outcome VA	6/6–6/18	41	25 (19–32)	33 (22–44)	8 (−3–19)	83	17 (12–22)	22 (17–26)	5 (−1–11)	71	24 (18–30)	47 (23–70)	23 (−1–47)
	<6/18	24	16 (13–20)	24 (17–31)	7 (0–14)	16	14 (10–17)	29 (10–48)	16 (−3–34)	28	23 (18–29)	40 (28–51)	16 (5–28)
	p-value		0.11	0.40	0.95		0.65	0.43	0.17		0.58	0.51	0.62
Eyes operated	1	44	21 (16–25)	27 (20–35)	7 (0–13)	75	14 (12–16)	23 (17–29)	9 (3–15)	67	24 (18–31)	36 (27–44)	11 (2–21)
	2	21	25 (15–34)	34 (17–50)	9 (−10–29)	24	24 (7–41)	23 (16–30)	−1 (−16–14)	30	21 (17–26)	66 (12–120)	45 (−9–99)
	p-value		0.56	0.37	0.80		0.11	0.66	0.21		0.66	0.36	0.22

We also explored how PCE was allocated and how this allocation changed over time. The largest proportion of PCE was spent on food both at baseline and at follow-up in all three countries. In the wealthier operated cases (i.e. above the median PCE at baseline) the largest increase in expenditure from baseline to follow up was on non-food items, so that the proportion of expenditure on food fell. In contrast among the poorer half (below the median PCE at baseline) most of the increased spending was on food items. As a result, at follow up there was little difference in expenditure on food between the poorer and wealthier operated cases.

## Discussion

This intervention study conducted in Kenya, Bangladesh and the Philippines showed that one year after cataract surgery PCE increased significantly among operated cases so that it was no longer lower than controls, while assets remained largely unchanged and self-rated wealth improved in Kenya and the Philippines. Overall PCE increased between 36% and 88% in the three countries. These gains were apparent in different socio-demographic and ophthalmic groups, and did not vary by ophthalmic characteristics. Gains in PCE were most noticeable in the most vulnerable groups, that is, those who were poorer, older, female or unmarried.

These data can provide further insight into the association between poverty and cataract. In this study, cases had lower levels of education than controls and thereby potentially more early life poverty. In addition, cost was cited as the main barrier to uptake of cataract surgery in this study [14]–[16], as it is in other studies [21]. This confirms the “selection effect” whereby poorer people are more likely to become blind because they are less likely to have their cataract treated [22]. Together, these data suggest that poverty causes blindness.

The data also suggest that cataract blindness may cause poverty. In this study the association between poverty and cataract at baseline persisted after adjustment for health, markers of early-life wealth (e.g. education) and social support, potentially suggesting an additional direct effect of cataract on poverty. One possible explanatory route for cases being poorer than controls at baseline is the impact of visual impairment on productivity. A companion paper from this study showed that cases were less likely to participate in productive activities (i.e. paid work and non-market activities) compared to controls at baseline [10]. Cataract blindness may also have resulted in restrictions of productivity of other household members as almost half of cases in Bangladesh and a quarter of cases in Kenya and the Philippines reported receiving assistance from household members at baseline (compared to <10% of controls) [10]. Furthermore, the time-use data suggested that after surgery the operated cases were significantly more likely to be involved in productive activities and spent on average 1–2 hours more on these activities in each setting. At the same time the frequency of reported assistance with activities more than halved in each setting [12]. The increase in PCE and consequent reduction in poverty that was evident after cataract surgery could therefore potentially be explained by increases in productivity of cases after surgery. This lends empirical support to the argument that blindness contributes to poverty and provides further evidence for the cyclical link between poverty and disability.

Other studies, though sparse, are consistent with our findings. The cross-sectional association between poverty and blindness has also been demonstrated in Pakistan [2], India [1], and Cambodia [3]. Longitudinal studies have demonstrated a reduction in employment and productivity associated with the onset of disability and that this is reversed if disability is alleviated [22]. A survey of patients in India also demonstrated the impact of blindness on loss of jobs, and found that most people who had lost their job as a result of blindness subsequently regained employment after cataract surgery, with consequent increased productivity [4]. The selection effect, whereby poor people are more likely to become disabled was clearly demonstrated using longitudinal data in the UK [22], and in Ireland [23].

There were a number of limitations to the study. Recall bias was possible since operated cases were generally very satisfied with their surgery. However, smaller changes were seen for asset scores or self-rated wealth, the latter being arguably the most subjective and therefore vulnerable to recall bias. Furthermore, the change in allocation of expenditure followed a meaningful pattern as expenditure on food did not increase among households that were richer at baseline, whereas expenditure on food increased among the poorer households. This supports Engel's law that states that as income rises the proportion of income spent on food falls [24], so that we would expect to see greater gains in expenditure on food among the poorer compared to the wealthier households as was the case in this study. PCE among controls was similar at baseline and follow-up providing support for cataract surgery being the key causal factor in the changes among operated cases.

Improvements in PCE at follow-up were observed among un-operated cases despite having received no intervention. The increase in PCE was of a similar magnitude in Kenya for the two case types, in Bangladesh it was about half the amount for the un-operated cases as for the operated cases, and in the Philippines it was about one third of the level in the un-operated cases as in the operated cases. The multivariable analyses showed few differences for poverty variables between operated and un-operated cases at baseline or follow-up in Kenya, while in Bangladesh and the Philippines there was some indication that the operated cases received benefits by follow-up in terms of PCE increases compared to the un-operated cases. However, the sample size was not powered to detect differences between operated and un-operated cases. It is not clear why PCE increased among un-operated cases, and similar changes were not observed in vision-related or generic quality of life [12], or participation in productive activities [13]. One possible explanation is that the household members of the un-operated cases, and the cases themselves, adapted over time so that the productivity constraints on the carer(s) was reduced.

PCE showed a greater change after surgery compared to the other measures of poverty (assets and self-rated wealth). PCE is a short-term measure of wealth and can change rapidly as the circumstances of the household change (e.g. a member becomes involved in paid employment or is able to cultivate land). In contrast, assets and self-rated wealth are longer term measures of poverty, as it takes time to accumulate assets or to alter perception of household wealth. Assets and self-perceived wealth are therefore less responsive to change compared to PCE and therefore one year of follow-up may have been insufficient to observe an impact of cataract surgery on these measures.

We did not consider economies of scale or use equivalence scales, because there is no widely accepted alternative to the simple equal-sharing convention. However, the majority of expenditure was on food which does not allow for economies of scale and the case and control households were of similar sizes in the three settings [11]. We only measured economic poverty, and did not consider social participation or inclusion or other aspects of wellbeing, although quality of life and activities are subjects of other analyses from this study [8]–[10].

Finally, the uptake of surgery was lower than anticipated. As a consequence there are concerns that we had insufficient power to detect the associations (particularly in Kenya). There were few differences in poverty or socio-demographic characteristics between operated and un-operated cases limiting the potential impact on external validity. The exception was that un-operated cases were older than the operated cases. It is therefore possible that if more of the un-operated cases had undergone surgery and been included at the follow up, average increases in PCE may have been slightly smaller. We also had to include two different case types although, the socio-demographic characteristics of cases identified through the RAAB was very similar to those identified through population-based case finding indicating that this would have contributed little to selection bias.

In terms of strengths, this was the first study to assess longitudinally the impact of cataract surgery on poverty in LMICs. It was large and allowed comparisons across three international settings. We selected population-based cases rather than cases presenting at the clinic in order to reduce selection bias and improve generalisability of our findings. We used the same standard questionnaires at baseline and follow-up in the three settings. We assessed poverty using three complementary indices, which included short-term measure which is responsive to change (PCE), as well as long-term measures (asset) and self-perceptions of wealth. PCE is generally believed to be a good indicator of current standard of living, and is more accurately recalled than income [7].

In conclusion, this study showed for the first time that cataract surgery can contribute to poverty alleviation, particularly among the most vulnerable members of society. Almost all our participants were living on less than $1 per day, and so are target of the first MDG. This study provides strength to the argument that a focus on blindness and potentially disability more broadly is an important step in achieving the MDGs.

## References

[1] Dandona L, Dandona R, Srinivas M, Giridhar P, Vilas K (2001). Blindness in the Indian state of Andhra Pradesh.. Invest Ophthalmol Vis Sci.

[2] Gilbert CE, Shah SP, Jadoon MZ, Bourne R, Dineen B (2008). Poverty and blindness in Pakistan: results from the Pakistan national blindness and visual impairment survey.. Bmj.

[3] Zimmer Z (2008). Poverty, wealth inequality and health among older adults in rural Cambodia.. Soc Sci Med.

[4] Javitt JC, Jamison DT, Mosley WH, Measham AR, Bobadilla JL (1993). Cataract.. Disease control priorities in developing countries.

[5] Resnikoff S, Pascolini D, Mariotti SP, Pokharel GP (2008). Global magnitude of visual impairment caused by uncorrected refractive errors in 2004.. Bull World Health Organ.

[6] Baltussen R, Sylla M, Mariotti SP (2004). Cost-effectiveness analysis of cataract surgery: a global and regional analysis.. Bull World Health Organ.

[7] Braithwaite J, Mont D (2009). Disability and poverty: a survey of World Bank Poverty Assessments and implications.. European Journal of Disability Research.

[8] Polack S, Kuper H, Mathenge W, Fletcher A, Foster A (2007). Cataract visual impairment and quality of life in a Kenyan population.. Br J Ophthalmol.

[9] Polack S, Kuper H, Wadud Z, Fletcher A, Foster A (2008). Quality of life and visual impairment from cataract in Satkhira district, Bangladesh.. Br J Ophthalmol.

[10] Polack S, Kuper H, Eusebio C, Mathenge W, Wadud Z (2008). The impact of cataract on time-use: results from a population based case-control study in Kenya, the Philippines and Bangladesh.. Ophthalmic Epidemiol.

[11] Kuper H, Polack S, Eusebio C, Mathenge W, Wadud Z (2008). A case-control study to assess the relationship between poverty and visual impairment from cataract in Kenya, the Philippines, and Bangladesh.. PLoS Med.

[12] Polack S, Eusebio C, Mathenge W, Wadud Z, Mamun R (In press). The impact of cataract surgery on health related quality of life in Kenya, Bangladesh and The Philippines.. Ophthalmic Epidemiol.

[13] Polack S, Eusebio C, Mathenge W, Wadud Z, Mamun R (2010). The impact of cataract surgery on time-use: results from a longitudinal study in Kenya, Bangladesh and The Philippines.. PLoS One.

[14] Mathenge W, Kuper H, Polack S, Onyango O, Nyaga G (2007). Rapid Assessment of Avoidable Blindness in Nakuru District, Kenya.. Ophthalmology.

[15] Wadud Z, Kuper H, Polack S, Lindfield R, Rashid Akm M (2006). Rapid Assessment of Avoidable Blindness and Needs Assessment of Cataract Surgical Services in Satkhira District, Bangladesh.. Br J Ophthalmol.

[16] Eusebio C, Kuper H, Polack S, Enconado J, Tongson N (2007). Rapid assessment of avoidable blindness in Negros Island and Antique District, Philippines.. Br J Ophthalmol.

[17] Turner AG, Magnani RJ, Shuaib M (1996). A not quite as quick but much cleaner alternative to the Expanded Programme on Immunization (EPI) Cluster Survey design.. Int J Epidemiol.

[18] Grosh M, Glewwe P (2000). Designing household survey questionnaires for developing countries. Lessons from 15 years of the living standards measurement study.

[19] Filmer D, Pritchett L (2001). Estimating wealth effects without expenditure data - or tears. An application to educational enrollment in states of India.. Demography.

[20] Rabin R, de Charro F (2001). EQ-5D: a measure of health status from the EuroQol Group.. Ann Med.

[21] Lewallen S, Courtright P (2000). Recognising and reducing barriers to cataract surgery.. Journal of community eye health.

[22] Jenkins SP, Rigg JA (2004). Disability and disadvantage: selection, onset, and duration effects.. Journal of Social Policy.

[23] Gannon B, Nolan B (2007). The impact of disability transitions on social inclusion.. Soc Sci Med.

[24] Engel E (1857). Die Productions und Consumptions-verhaltnisse des Königsreiche Sachsen.. Zeitschrift des Statistischen Bureaus des Königlich Sächsischen Ministerium des Inneren.

